# Psychosocial Factors Affecting Smoking Cessation Among People Living With Schizophrenia: A Lived Experience Lens

**DOI:** 10.3389/fpsyt.2019.00565

**Published:** 2019-08-15

**Authors:** Nadine Cocks, Lisa Brophy, Catherine Segan, Anthony Stratford, Simon Jones, David Castle

**Affiliations:** ^1^Research and Advocacy, Mind Australia Limited, Heidelberg, VIC, Australia; ^2^Melbourne School of Population and Global Health, The University of Melbourne, Melbourne, VIC, Australia; ^3^School of Allied Health, Human Services and Sport, College of Science, Health and Engineering, La Trobe University, Bundoora, VIC, Australia; ^4^Quit Victoria, Cancer Council Victoria, Melbourne, VIC, Australia; ^5^Department of Psychiatry, University of Melbourne, Melbourne, VIC, Australia; ^6^Department of Psychiatry, St Vincent&rsquo;s Hospital Melbourne, Fitzroy, VIC, Australia

**Keywords:** smoking, schizophrenia, recovery, lived experience, stigma, marginalization, peers

## Abstract

**Introduction:** People living with schizophrenia smoke at much higher rates than the general population, and find it more difficult to quit. To date, lived experience has received little attention from researchers. Personal recovery perspectives may generate further insights into established psychosocial barriers and enablers of smoking cessation.

**Methods and Results:** A lived experience account is provided by one of our authors that places the current evidence in context, and highlights the role of marginalization and stigma in reinforcing smoking. Key concepts from the personal recovery paradigm, such as connectedness, hope, and empowerment are discussed. The relevance of these factors and the value of shared lived experience in challenging stigma, marginalization, and low expectations demonstrates the contribution that peer support can offer to support smoking cessation.

**Conclusions:** Recovery-oriented approaches when integrated with existing evidence-based treatments designed to meet the needs of people living with schizophrenia have potential to improve outcomes by helping to take a more holistic approach to break down barriers and facilitate increased uptake of treatment and support. Further research to evaluate the effectiveness of integrated approaches is warranted.

## Introduction

People living with schizophrenia are at least five times more likely than people in the general population to smoke tobacco and are less likely to quit successfully (1). Moreover, a person living with schizophrenia consumes more cigarettes per day with a greater preference for unfiltered, high nicotine and high tar cigarettes than does a smoker in the general population (2, 3). Overrepresentation of social risk factors for smoking such as social norms that support smoking, social and economic disadvantage, unemployment, and alcohol and substance misuse contribute to these high smoking rates. As smoking kills around one in two long-term users, it makes a significant contribution to the premature mortality observed in people living with schizophrenia (4). Recent studies have found that there is up to 20 years reduction in life expectancy associated with a diagnosis of schizophrenia, largely due to smoking-related diseases (5).

A 2010 Australian national survey of people living with psychotic illness reported a smoking rate of 67%, unchanged from the previous survey conducted 15 years prior and contrasting with a 7% decline in smoking in the general population over the same period. Despite these high smoking rates, studies involving people living with schizophrenia recruited from a range of mental health service settings indicate that they are as motivated to quit smoking as other people in the general community (6). This motivation to quit is not necessarily dependent on level of symptoms. It has been found that people with more symptoms related to their mental illness have been more highly motivated to stop smoking than those with less (7).

Understanding the reasons why people living with schizophrenia continue to smoke at such high rates despite increasing efforts at the general population level to support smoking cessation is vital to informing the development of more nuanced and effective policy and interventions. The reasons why people living with schizophrenia continue to smoke may be not dissimilar to the general population. People living with schizophrenia are more likely to experience established barriers to cessation, such as higher levels of addiction, greater likelihood of living with smokers, more prosmoking social norms, greater financial stress, and increased depression and anxiety. In addition, they are often less likely to be formally supported in their efforts to stop smoking and are also less likely to be able to afford nicotine replacement products. Ongoing stigma, discrimination, inequality, and social exclusion are also considered to play a part in both why people smoke and add to the challenges of quitting (8, 9).

It is not the purpose of this paper to further stigmatize people living with schizophrenia by rarefying their experiences around smoking. Alternatively, our aim is to try to understand the challenges this group faces in quitting and how best to offer them support to address their smoking. Regular tobacco use has a devastating impact not only on physical health but also on psychosocial recovery as it entrenches people in a cycle of financial, social, and emotional disadvantage (10). Thus, there is a current imperative to reduce the very high rates of smoking among people living with schizophrenia and other severe mental health conditions even though there are significant challenges associated with both supporting individuals to quit and making the services they access more responsive to this issue.

While considerable progress has been made in introducing smoke-free environments into mental health settings, embedding smoking cessation care, such as brief advice that links smokers to effective behavioral and pharmacological treatments, remains limited. The role of societal factors, particularly stigma and discrimination, highlights the need to address the attitudes of some health professionals and other service providers who have been commonly found to not support the efforts of disadvantaged and marginalized people to quit smoking (9).

The introduction of a lived experience ‘lens’ on this situation presents an opportunity to include a recovery perspective on the question of both why people living with schizophrenia smoke and how smoking cessation programs could adopt a more recovery-oriented approach. Experiential knowledge, such as first-person experience, has the potential to extend—and critique—professional knowledge that has been derived mainly from the traditional hierarchy of evidence. It gives voice to those who are experts through their lived experience and enables a shift in power, bringing greater respect to the value of subjectivity (11). Hence its relevance to a recovery orientation that emphasizes concepts such as empowerment and the value of peer support (12, 13).

## Background

The Cancer Council of Victoria (Australia) presents a comprehensive review of smoking and health issues in Australia (14). This report synthesizes data and information in relation to Australia’s tobacco control program including smoking consumption and trends, the health effects of tobacco use, addiction, smoking cessation programs, smoking and social disadvantage, and smoking and public education campaigns. The following reflects a number of findings presented in this report.

### Excess Mortality and Schizophrenia

The problem of people living with schizophrenia dying much earlier than expected is well established (4). Although premature death has commonly been explained as being the result of unnatural causes such as suicide and violence, it is now understood that premature death from natural causes, such as heart disease and cancer, is “at least as important a source of the excess mortality in mental disorder as death from unnatural causes” (p. 51) (15).

The life expectancy of people living with schizophrenia is approximately 20% shorter than that of the general population (16) and there is evidence that this gap widening over time (4, 17). The mortality risks for people with schizophrenia has been compared to the impact of heavy smoking (17). Moderate to heavy smoking can result in an 8 to 10 year loss in life expectancy; however, some recent studies have found that there is up to 20 years reduction in life expectancy associated with a diagnosis of schizophrenia, largely due to smoking-related diseases (5). Smoking is just one of the indicators of poor health among people living with schizophrenia and what is required is a more holistic approach to improving physical health (18). People living with schizophrenia are also at higher risk of other serious health challenges including poor dental health, obesity, diabetes, hypertension, and cardiovascular (CV) disease and about half of total deaths in people living with schizophrenia can be attributed to these smoking related health conditions (14, 19–21) (22). Comorbidity with other psychiatric disorders, including the mood and anxiety disorders, is common in schizophrenia (23) and these disorders are also associated with smoking (24). Also, people living with schizophrenia may also experience problems with alcohol and illicit drugs at higher rates than the general community (25) and this may also be linked to increased smoking and decreased mortality. Therefore, supporting people to quit smoking is an important strategy to deal with this significant and potentially increasing mortality gap, especially where cessation treatments also encourage the adoption of a healthy lifestyle that may concurrently address other physical health risk factors that people living with schizophrenia commonly experience. Lum et al. (8) found that for people living with schizophrenia, health concerns are the highest reason to quit, and in some cases, the perceived smoking related health implications are the most negative consequence of smoking.

### Survey of High Impact Psychosis: SHIP (2010)

Based on the 2010 Australian National Survey of High Impact Psychoses (SHIP), Cooper et al. (26) present the patterns of smoking for Australians living with a psychotic illness and relationship of smoking to other health, psychosocial, and demographic characteristics (26). They concluded that the prevalence of smoking for people living with mental illness is not changing, in contrast to the mainstream Australian population, and more research is needed to further understand what other barriers may exist for this cohort upon which targeted interventions may be based. In a discussion with a group of people living with psychosis about findings from the Australian Study of Low Prevalence (Psychotic) Disorders conducted in 1997, participants expressed considerable feelings of hopelessness and lack of social connection that they linked to smoking in comments such as *‘What is there to stop for*?” and “*When you wake up in the morning, what else is there*?” (p. 11) (27). Hence it may be that in the time between surveys efforts to support smoking cessation among people living with high impact psychosis have not adequately addressed issues of marginalization, lack of social inclusion, and minimal meaningful occupation.

## Barriers to Smoking Cessation—Psychosocial Perspectives

Through their systematic review Lum et al. (8) examined the barriers and facilitators to smoking cessation in people living with schizophrenia. They note that there is much more research into pharmacological smoking cessation interventions for people with schizophrenia and little research examining psychosocial interventions. The review focused on psychosocial factors in order to inform the development of psychosocial interventions (rather than review psychosocial interventions per se). However, in identifying psychosocial smoking cessation interventions, such as psychoeducation, motivational interviewing, and cognitive behavioral therapy, they observe that very few randomized controlled trials have been conducted with people living with schizophrenia or examination of their underlying theories. This is in contrast to the neurobiological links that have been identified to inform pharmacological treatment.

Lum et al. (8) found that cravings and addiction was the most commonly cited barrier to smoking cessation in people living with schizophrenia, and in some cases perceived or actual cravings associated with smoking cessation was “significantly higher among people living with mental illness” (p. 5). Negative affect was also identified as a major barrier to cessation. Smokers report using smoking as a tool to alleviate symptoms of anxiety and depression and to manage stress and boredom and are often concerned how they might cope without smoking, including that they may become unwell again [Lawn et al. (28) in Ref. (8)]. This fear of increased negative affect, together with the fact that negative affect is also a nicotine withdrawal symptom, are barriers to initiating quit attempts. In addition, once people make a quit attempt, increased negative affect is a strong predictor of smoking relapse.

People living with schizophrenia are at high risk of social exclusion and boredom in their everyday lives and smoking has been found to provide some opportunity for social affiliation and inclusion (8, 14). In relation to social barriers, Trainor and Leavey (29) refer to smoking enabling consumers to fit in and feel included, relieving loneliness and alleviating stigma. Lum et al. (8) identified social reasons why people living with schizophrenia smoke and are unlikely to quit. Consumers use smoking to improve social functioning and fear they will be socially ostracized if they give up (8).

There is also a historical and environmental context that potentially explains high rates of smoking among people with schizophrenia (14). High rates of institutionalization in the past were associated with an institutional ethos in which smoking was often central to daily activities. Despite deinstitutionalization many argue that the culture and traditions of institutions often remain in the approach to care that predominantly now occurs in the community. Hall and Prochaska (6) refer to the smoking culture in mental health settings which is based on the priority of mental health treatment, ambivalence of the health effects of smoking, and belief that psychiatric patients are unable to quit. In one study, reviewed by Lum et al. (8), 83% of psychiatric inpatients with schizophrenia believed that visitors should be allowed to smoke with patients (30). In this culture, cigarettes have been provided by clinicians as a reward and to ensure compliance. In agreement, Lawn (31) identifies cigarettes as ‘currency’ for psychiatric patients in mental health institutions. In this environment, where smoking is supported and promoted by staff and patients alike, escaping from this culture and quitting is extremely difficult (14).

Hahn et al. (32) investigated the rates of smoking for people living with mental illness in a disadvantaged area of Adelaide in South Australia and identified social barriers that increase smoking rates in the community (32). They found ‘strikingly’ high rates of smoking for both men and women within their environment of social disadvantage marked by high unemployment, low rates of education, high rates of public housing and poorer health outcomes. In their view, smoking cessation programs for this cohort cannot be provided in isolation from other supports in the community. In parallel, other measures to promote employment, physical activity, health, and well-being and social engagement in the community are more likely to help people quit.

### Access to Smoking Cessation Support

Lum et al. (8) found that while 80% of people living with schizophrenia receive advice from health professionals to quit, the “5 A’s” (ask, advise, assess, assist, arrange) smoking cessation strategy has not been effectively implemented—with practitioners citing lack of interest from patients, too many demands (including time) on staff, and skepticism of the program as barriers. Lack of smoking cessation programs in hospitals is seen as a barrier to quitting and nicotine replacement therapy (NRT) is seen as negative, unhealthy with unwanted side effects, and unnecessary. Generally, staff support liberal smoking rules in psychiatric inpatient units (8). A high proportion of participants in studies reviewed find it too difficult to quit especially when seeing other patients smoke and when staff do not set a good example. There appears to be persistent ‘myths’ associated with smoking and schizophrenia that may entrench a lack of support for people to quit. In an Australian study, Wye et al. (33) investigated the implementation of smoking bans in psychiatric inpatient services. While the researchers note that general hospitals have successfully transitioned to smoke-free environments, they also found that there were ongoing challenges in implementing change in clinical mental health settings, even though there is widespread support for smoking bans among staff. Staff presented concerns in relation to perceived patient aggression and the lack of capacity and organizational support for change. The researchers identified that more cultural and systematic change, strong leadership, and staff training and support is necessary to help those with mental illness quit and alleviate their health inequalities associated with smoking.

In summary, people living with schizophrenia face significant challenges and barriers to quitting smoking (29). While consumers acknowledge the negative health consequences associated with smoking, they find little support from mental health practitioners, and cessation programs, especially psychosocial interventions to help them quit (8, 34).

## The Lived Experience of One of Our Authors

### A Personal Reflection on Reasons for Smoking and What Helps

Through exploring some of the reasons why people with mental health issues smoke, and the difficulties they experience with smoking cessation, from a lived experience perspective, my smoking experiences highlight reasons to do with loss of connection, isolation, stigma, boredom, loss of identity, and a desire to belong as my story explains:

I began smoking regularly during my first psychotic episode. Before that, I was a smoker just on occasions such as parties and usually associated with drinking. Something, however, happened when I became ill. I was frightened, overwhelmed and scared. I seemed not to be able to control the thoughts in my head and the smoking became my comfort. The rush of nicotine somehow made me feel better and though I was breathing in deadly toxins and poisons, smoking actually forced me to breathe deep breaths. It also enabled me to structure my day around smoking breaks and forced me to spend some time outdoors as I had a rule to never smoke indoors.

Smoking also had the benefit of making me feel like I belonged. I would love to smoke with other smokers (usually with mental health issues) and this gave me a sense of camaraderie. We felt like we were part of a group and the smoking places became ours. This also played a part in combatting stigma and made each of us feel included.

I would regularly smoke with coffee and the cigarettes combined with the coffee helped combat some of the sedating effects of the medication. Together they gave me a ‘lift” and it soon became a ritual I practised often.

I believe I was self-medicating, that is, using the cigarettes to somehow limit the effect of the illness. However, I was not only smoking to achieve this outcome, smoking also had other benefits. It relieved me of boredom and a sense of things being out of control, and I became hooked as much on the nicotine, as the ritual of rolling my own cigarettes—a ritual I became very skilled at.

I tried several times to quit with the help sometimes of Quitline telephone support services but always returned to smoking.

It wasn’t until the graphic ad campaign of the 2000’s that I became shocked and frightened about what the cigarettes were doing to my body that really helped me to finally quit. I did it with the help of nicotine-replacement and the Quitline telephone support service and have been smoke free for years.

Since I quit, I have been able to focus much more on my health. Being able to breathe properly made exercise easier and more enjoyable and has added benefits of giving me an endorphin rush which has replaced the cigarette rush. I was also able to practice mindfulness, yoga and other activities where concentrating on the breath is so important. I have developed an intense appreciation of nature and the natural environment. I am able to appreciate the outdoors without a cigarette and really connect and appreciate my environment without the crutch of a cigarette. My mental health improved greatly and I was generally a lot calmer and more relaxed. The money saved from quitting has contributed to me being able to travel overseas and experience many diverse cultures.

It was very important for me to develop alternative strategies to replace smoking and also to work on my protective behaviours to help manage some of the withdrawal effects and changes in mood. What I found helpful was a holistic approach to improving my physical health but I found this only happened when I quit smoking.

### Connecting Lived Experience With the Literature

As this reflection describes, smoking helps address some of the psychosocial dimensions of people’s experience with serious mental illness such as lack of connection, social isolation, stigma and loss of identity. Schizophrenia is a very stigmatizing condition and therefore these dimensions are even more pronounced.

The reasons why people living with schizophrenia smoke from a lived experience perspective include stigma and marginalization and a sense that hope or a positive sense for the future is lacking. However, even though people living with schizophrenia face significant stigma and discrimination, their voices on stigma are largely underrepresented in the literature (35). Hence the importance of future research that privileges the voices of people with lived experience to enable greater recognition of these factors.

The current literature, and this personal story, support the perspective that smoking in groups can make people feel they are not alone and cigarettes play a role in helping people bond and develop a sense of camaraderie. Conversely, with current restrictions regarding public places where people can smoke, some smokers experience isolation by their smoking behavior and share a sense of ‘camaraderie in exile’ with other fellow smokers in designated smoking areas (36). Smokers report stigmatizing attitudes from the community when smoking in public. Alternatively for some smokers there might be an experience of having ‘permission’ to smoke when they see others smoking in public (36). As cigarette smoking is banned in many places and smoking is more prevalent in groups of people experiencing mental ill health, people living with schizophrenia are being further stigmatized and marginalized for being smokers. Indeed, Lum et al. (8) concluded that social pressure to quit smoking is reportedly higher among people living with schizophrenia. This in turn impacts on their self-esteem and sense of self-worth but also paradoxically, or as an unintended consequence, creates a group identity or a subgroup of belonging.

Smoking with groups of smokers may negate the stigma that people living with schizophrenia face through having a sense of group identity. Illness identity is another factor that may impact on the hope and self-esteem of people who smoke and are living with a mental illness (37). This illness identity can be pervasive and contribute to feelings of hopelessness, and not having a positive sense of the future.

Resilence may also play a part in why people living with schizophrenia smoke and others do not. Lawn et al. (38) outline a resilience construct which may explain why some people are able to draw on protective behaviors that act as a buffer against taking up smoking.

Regardless of negative factors in people’s lives some people are able to draw on resources to help them deal with challenging experiences and situations. This is described by Lawn et al. (38) as the resilience construct. From a critical view of the literature, Lawn et al. (38) propose that resilience be defined as “the interaction between the internal properties of the individual, and the set of external conditions, that allow individual adaptation, or resistance to different forms of adversity at different points in the life course” (p. 47) (38).

It could be that when a person is experiencing mental ill health their protective behaviors and resistance is low or lower than usual. Perhaps by helping people develop and learn alternative coping skills and strategies that sit within a recovery framework (discussed below), both people who are at risk of smoking and smokers wanting to quit could benefit.

Most people are not offered best practice treatment and there is a need to offer support and treatment to all smokers, not just those interested in stopping smoking (39). Taking into account the discussion above, best practice treatment would recognize that smoking cessation rates are maximized when brief intervention from a health or support worker links people living with schizophrenia to both a multisession specialist behavioral intervention [from, for example, Quitline telephone support (40) or a group course] plus pharmacotherapy (nicotine products or cessation medications).

## The Recovery Framework

A recovery framework is defined by the *National Framework for Recovery-Oriented Mental Health Services* (41) as providing holistic and ‘person first’ services that supports personal recovery, an organizational commitment, workforce development, and action on social inclusion and the social determinants of health.

Fundamental to any recovery framework are the elements of hope, social connection and empowerment (42). In championing these elements of recovery; marginalization, stigma, and social disconnection can be addressed. Corrigan et al. (43) outline the ways stigma operates through fear and exclusion, authoritarianism, and benevolence, and they suggest that stigma can be tackled by protesting for people with mental illness, educating the community and increasing the amount of contact people have with other people who live with mental health issues. Through people with mental health issues being supported to empower themselves, regain a sense of social connection, and hope for the future they can regain autonomy and control of their lives.

If people living with mental health issues, and more specifically schizophrenia, are supported to develop alternate coping skills, resilience, and strategies for smoking cessation within a recovery framework that acknowledges the impact of stigma and discrimination and encourages hope, social connection, and empowerment, they may have a better chance in quitting smoking.

Malpass and Higgs (44) have hypothesized that by using smoking to cope with their mental ill health, alternative coping skills and strategies may not be explored, maintaining their mental ill health and smoking behaviors. However, the act of quitting smoking itself is very empowering and can increase confidence and self-esteem for the person who has quit. Quitting can also lead to an increased interest and focus on physical health from a holistic perspective and should be seen as an important aspect of the recovery journey supported with recovery oriented smoking cessation strategies.

Smoking is just one of many risk factors for poor health that are more common among people with schizophrenia. Integrating recovery-oriented approaches with existing evidence-based treatments for individual risk factors can provide a more holistic approach (18). As the consumer movement has moved toward rearticulating recovery in terms of well-being, consumers are now expecting services and treatments to be both holistic and person centred. It may be useful to view smoking cessation as one of the many aspects of recovery and well-being that consumers engage with.

In the lived experience example presented above, the author developed alternate coping strategies such as yoga, mindfulness, and getting outdoors to support both the quit attempt and also to put in place some long lasting permanent strategies for health and well-being. Such strategies can be combined with supports such as Quitline telephone support (40) and pharmacotherapy to increase the chances of quitting successfully thus experiencing recovery-oriented cessation support

Significant reductions or cessation of tobacco smoking provides positive opportunities for people to achieve their individual social and economic goals and improve both their physical and mental health. Mental health settings urgently require a recovery-oriented approach to smoking that is flexible, evidence based and sustainable.

## Recommendations

As indicated in the personal story above, evidence-based treatments work. In this situation best practice treatment that includes a combination of a multisession behavioral intervention (e.g., Quitline telephone support) plus NRT made an important contribution to quitting. A major challenge remains to make routine the delivery of brief smoking cessation advice by mental health service staff, including peer workers, that proactively links people who smoke to these effective forms of help.

Incorporating a recovery orientation approach to smoking cessation treatment highlights the value of peer support. This requires the intentional use of lived experience in the support of others, and recognition that concepts like connectedness, hope, identity, meaning, and empowerment have an important role in supporting the efforts of people living with severe mental illness to quit (42).

Peer support models are being adopted in mental health contexts in many different ways including management, representation, advocacy, direct service, training, and research. Evidence suggests that peers can engage persons who have been difficult to reach and have not benefitted from traditional services and that peer workers can decrease the costly use of acute services like emergency room visits and hospitalizations while increasing the use of outpatient care. Furthermore, peer work can reduce demoralization and the use of alcohol, while increasing hope, empowerment, and self-care (13). Informal supporters and peer supporters have also been found to support smoking cessation in people living with schizophrenia and other serious mental illness (45–47). A further example of this peer led approach is the Quitlink randomized controlled trial of peer worker facilitated Quitline support plus combination NRT, for smokers receiving mental health services [Ref. (48) in this issue].

Multisession behavioral interventions that tailor to the needs of people living with schizophrenia include monitoring of nicotine withdrawal symptoms, many of which overlap with mental health symptoms, so this dually acts as mood monitoring that helps to distinguish nicotine withdrawal from a relapse of mental illness. Monitoring of medication side effects is also required as smoking cessation increases blood levels of some medications and of alcohol and caffeine (49). Helping individuals to identify triggers to smoking and building skills to use alternate coping strategies is a mainstay of smoking cessation treatment. For people living with schizophrenia a focus needs to be given to building skills to manage negative affect, given its role as a barrier to both making and sustaining quit attempts. Lum et al. (8) discuss the potential of CBT behavioral activation approaches to address negative affect. Many mood management strategies can dually act as smoking cessation strategies. Building smoking refusal skills is also critical as is instituting alternative ways to feel rewarded to help reduce feelings of deprivation following smoking cessation (49–51).

Relapse is a common experience that needs to be normalized, recognizing that cessation may require multiple attempts to quit. The role of higher doses of NRT may also need to be considered as well as extended use of NRT or other cessation pharmacotherapies to prevent relapse. Feedback to the person’s treatment team about their progress is recommended to facilitate coordinated care.

Supporting people living with schizophrenia to quit also requires system change including increased training for staff, and policies and procedures that embed smoking cessation brief interventions that link people to multisession behavioral support, and availability of NRT to address nicotine dependence. Smoke-free inpatient experiences, in particular those that provide NRT at sufficient levels to actively manage nicotine withdrawal provide important smoke-free experiences that need to be further built on, for example by routinely offering on discharge further cessation pharmacotherapy and enrolment in multisession behavioral treatment by telephone (52).

## Conclusion

People living with schizophrenia want to quit smoking as much as people with other mental health issues, and the general community, but cessation rates remain low and this is contributing to significant physical health problems and premature death. Quitting smoking can lead to greater consideration of physical health, and as one of our authors describes, “being able to breathe properly enabled me to take up walking, yoga and mindfulness which in turn became powerful strategies to replace smoking”. The perspective of people with lived experience of mental ill health, smoking cessation and personal recovery further assists in thinking through how to address all the complex psychosocial factors we have discussed. This is a social justice issue that presents opportunities for people with lived experience to enhance the recovery orientation in current and future efforts to support smoking cessation. Integrating recovery-oriented approaches with existing evidence-based treatments designed to meet the needs of people living with schizophrenia have potential to improve outcomes by helping to take a more holistic approach to break down barriers and facilitate increased uptake of treatment and support. Further research to evaluate the effectiveness of integrated approaches is warranted.

The peer support model is based on shared responsibility, respect, and mutual understanding of what is helpful and together with the NRT and Quitline counselling, it is a potentially powerful strategy to support smoking cessation (53, 54). It is hoped that this approach will engage people with schizophrenia in successful quitting, thus enhancing their physical and mental health and psychosocial well-being and making an important and urgently needed contribution to reducing the mortality gap for those living with schizophrenia.

## Author Contributions

NC and LB led the writing of this paper and wrote core components. DC was senior author contributing oversight and core concepts. AS worked with NC on specialist content. SJ provided research assistance and summarized key literature. CS contributed specialist input and also wrote key section of the manuscript. LB prepared the paper for submission.

## Funding

This project is funded by the National Health and Medical Research Council (APP1139125). The funder had no role in study design, data collection and analysis, decision to publish, or preparation of the manuscript.

## Conflict of Interest Statement

The authors declare that the research was conducted in the absence of any commercial or financial relationships that could be construed as a potential conflict of interest.
